# A Holistic View of the Goto-Kakizaki Rat Immune System: Decreased Circulating Immune Markers in Non- Obese Type 2 Diabetes

**DOI:** 10.3389/fimmu.2022.896179

**Published:** 2022-05-23

**Authors:** Snehaa V. Seal, Mathilde Henry, Clémentine Pajot, Cyrielle Holuka, Danielle Bailbé, Jamileh Movassat, Muriel Darnaudéry, Jonathan D. Turner

**Affiliations:** ^1^Department of Infection and Immunity, Luxembourg Institute of Health (LIH), Esch-sur-Alzette, Luxembourg; ^2^Faculty of Science, Technology and Medicine, University of Luxembourg, Esch-sur-Alzette, Luxembourg; ^3^Institut National de Recherche Pour l'agriculture, l'alimentation et l'environnement (INRAE), Bordeaux Institut National Polytechnique (INP), NutriNeuro, Unité Mixte de Recherche (UMR) 1286, University of Bordeaux, Bordeaux, France; ^4^Université de Paris, Laboratoire B2PE (Biologie et Pathologie du Pancréas Endocrine), Unité BFA (Biologie Fonctionnelle et Adaptative), Centre National de la Recherche Scientifique -Unité Mixte de Recherche (CNRS UMR) 8251, Paris, France

**Keywords:** diabetes, obesity, cytokines, inflammation, microarrays, Goto-Kakizaki rats

## Abstract

Type-2 diabetes is a complex disorder that is now considered to have an immune component, with functional impairments in many immune cell types. Type-2 diabetes is often accompanied by comorbid obesity, which is associated with low grade inflammation. However,the immune status in Type-2 diabetes independent of obesity remains unclear. Goto-Kakizaki rats are a non-obese Type-2 diabetes model. The limited evidence available suggests that Goto-Kakizaki rats have a pro-inflammatory immune profile in pancreatic islets. Here we present a detailed overview of the adult Goto-Kakizaki rat immune system. Three converging lines of evidence: fewer pro-inflammatory cells, lower levels of circulating pro-inflammatory cytokines, and a clear downregulation of pro-inflammatory signalling in liver, muscle and adipose tissues indicate a limited pro-inflammatory baseline immune profile outside the pancreas. As Type-2 diabetes is frequently associated with obesity and adipocyte-released inflammatory mediators, the pro-inflammatory milieu seems not due to Type-2 diabetes *per se*; although this overall reduction of immune markers suggests marked immune dysfunction in Goto-Kakizaki rats.

## Introduction

Type-2 diabetes (T2D) is a complex disorder characterised by hyperglycaemia, insulin resistance (IR) and chronic inflammation of insulin target tissues. T2D is now also considered an inflammatory disease, affecting both innate and acquired immune systems, skewing them towards a pro-inflammatory phenotype [reviewed in (1)]. There is also growing evidence for autoimmune involvement in T2D overlapping with the pathophysiology of T1D (2). Inclusion of cellular autoimmunity with traditional diabetic parameters is now reflected by a number of diabetes sub-types that do not fit into T2D or T1D including type 1.5 diabetes mellitus or latent autoimmune diabetes of the adult (LADA) or the young (LADY) and double diabetes (mixed symptoms of T1D and T2D) (3, 4). This recalibration of diabetic phenotypes led us to re-examine the use of obese pre-clinical diabetic models, as these no longer adequately reflect the human clinical context (5). Most of the studies conducted on inflammation in T2D involved overweight or obese subjects and obesity is clearly associated with a low-grade chronic inflammation (6, 7). Since T2D is not always associated with obesity, it is essential to dissect the exact contributions of obesity and diabetes in the immune phenotype.

Although there are many well-known obese models of T2D, there are few non-obese models (8). Goto-Kakizaki (GK) rats are a non-obese model that present a pre-diabetic phase before becoming spontaneously diabetic, similar to the human T2D pathophysiology (9). However, despite the growing immune literature in human T2D, there is little data available on the GK rat immune system. Circulating white blood cell (WBC) levels have been reported to be unchanged in GK rats, although they are known to be biased towards a T_h_2 phenotype (10), have fewer B-cells, a higher IgM production and reduced monocyte-phagocytic activity (5, 11). Surprisingly, this phenotype is somewhat contrary to that observed in the Type 1 model “Diabetes-prone Biobreeding (DP-BB) rats” (5) and high-fat diet induced obesity in mice (12). In contrast, previous works have demonstrated a marked proinflammatory profile in pancreatic islets in GK animals, with macrophage infiltrations and upregulation of mRNA expression of several proinflammatory cytokines in this tissue (13, 14). Subsequently fibrosis develops within the islets which further alter their normal secretory function (14).

Given the potential for the GK rat model to represent non-obese and emerging diabetes subtypes, we used a wide-ranging rat immune system profiling panel (15) and multiplex cytokine panel to characterise adult, diabetic GK rats. By investigating levels of principal circulating immune cell subsets and plasma cytokines, we aim to provide a baseline immune profile to further study the role of immune system in the aetiology of T2D. Furthermore, we reanalysed previous transcriptional datasets (16) for the consequences of exposure to this cytokine milieu in the liver, adipose tissue and muscles.

## Materials and Methods

### Animals

GK rats (from B2PE, unit BFA, Diderot and CNRS, Paris colony) and control Wistar rats were bred in the conventional facility of Nutrineuro lab (INRA UMR1286, Bordeaux). Bodyweight and adiposity in animals from the colony have recently been described (17), and are a common finding in GK rats (9, 13, 16) although may depend on colony (18). Six months old male offspring were used (n=6 Wistar, n=7 GK). All experimental procedures were carried out in accordance with the European Union guidelines for the use of animals for experimental purposes (Council Directive 2010/63/EU) and the French guidelines (Directive 87/148, Ministère de l’Agriculture et de la Pêche – Apafis #16924). Animals were grouped-housed (n=3 per cage) in a 12 h/12 h light:dark cycle (lights on at 06:00 a.m.) at 22 ± 2°C with access to food (SAFE D113, Augy, France) and water ad libitum.

### Blood Sampling

Blood samples were collected by tail nick into EDTA-coated tubes. Samples were centrifuged (1800g, 4°C) for 10 minutes. Plasma was collected and stored at −80°C for cytokine or insulin assays. For flow cytometry, blood was mixed with Streck cell preservative (1:1 volume) (Biomedical Diagnostics, Antwerp, Belgium), stored and transported at 4°C.

### Flow Cytometry

Staining was performed on Streck-preserved samples as described in **Supplementary Materials**. Briefly, 200,000 events were recorded on an LSRFortessa (BD BioSciences, NJ, USA) and analysed using FlowJo (version 10.6.1, BD BioSciences, NJ, USA). A Streck-preservative optimised gating strategy (**Supplementary Figure 1**) was adapted from Fernandes et al. (15) and populations represented as a percentage of parent population frequency. Data was not available for 1 GK rat due to Streck preservative associated coagulation and failure of the staining.

### Plasma Cytokines, Glycaemia and Insulin Levels

Glucose, Insulin, and a panel of 27 plasma cytokines (Milliplex 27-plex kits; Eve Technologies, Calgary, Canada), were measured as described in **Supplementary Materials**.

### Microarray Re-Analysis

Wistar and GK rat microarray data were downloaded from NCBI (GSE13271) for adipose tissue, gastrocnemius muscle, and liver from 20-week-old normal-diet fed animals (16). Raw data were normalised, a linear model fitted, and differential gene expression calculated using limma (version 3.46.0) for genes expressed above a threshold (100) in all 10 samples per comparison using genefilter (version1.54.2).

### Statistical Analysis

All statistical analyses were performed using Graphpad Prism (version 8.2.0). Unless otherwise stated the unpaired Student’s t-tests were used to compare normally distributed data from GK and Wistar rats when the variance was equal between the groups. Welch’s t-test was used for normally distributed data with unequal variance between the groups, and the non-parametric unpaired Mann-Whitney test was used for non-normally distributed data. All data are the result of two biologically independent experiments. Pearson correlations were performed on the complete dataset and were used to determine the associations between immune cells profile and plasma cytokine levels. Microarray and pathway reanalysis was performed in R (version 4.0.2). Venn Diagrams were generated with “VennDiagram” (version 1.6.20). Statistical significance was set at p < 0.05. Data are expressed as means ± SEM.

## Results

### GK Rats Show a Limited Pro-Inflammatory Immune-Cell Profile

**Figure 1:**
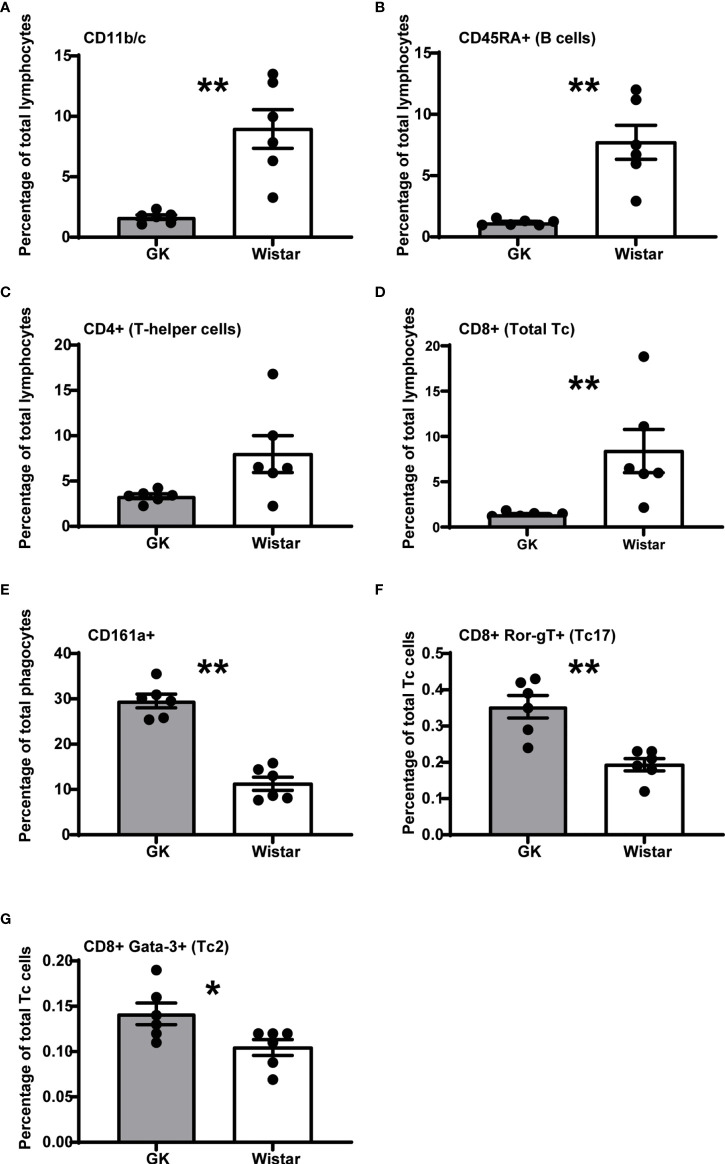
The diabetic milieu majorly alters the GK immune system. Significantly affected immune cells (p<0.05), expressed as percentage of parent population frequency are, **(A)** CD11b/c+ phagocytes (macrophages, monocytes, dendritic cells, granulocytes), **(B)** CD45RA+ B cells, **(C)** CD4+ T-helper cells, **(D)** CD8+ T-cytotoxic cells **(E)** CD161a+ NK cells, T-cell subsets, activated monocytes, and dendritic cells **(F)** CD8+ RORγT+ T_c_17 cells **(G)** GATA3+ T_c_2 cells. However, the T-helper cells narrowly missed the significance threshold (p = 0.0649). Data are mean +/- SEM; individual animals shown as dots. Grey bars: GK rats (n = 6); white bars, Wistar rats. (n = 6).

The numbers of circulating CD11b/c+ and CD45RA+ (B cells) cells in GK rats was ~2.5-fold (p=0.0022, Mann-Whitney test) and ~4-fold (p=0.0022, Mann-Whitney test) lower respectively, compared to Wistar rats (**Figures 1A, B**) as previously reported (10, 11). This suggests a decrease in the frequency of phagocytes such as macrophages, monocytes, dendritic cells, granulocytes and B-cells respectively.

GK rats have also been reported to have an increased frequency of total CD3+ T cells (5, 10, 11), although we did not observe this in our study (data not shown). The CD4+ T_h_ cells had a decreasing trend in the GK rats (~2.5-fold), compared to Wistar rats (p=0.0649, Mann-Whitney test, **Figure 1C**). This was in line with Huda et al., who reported a decreasing naive CD4+ T_h_ cell trend in diabetics (19).

We observed a significant ~5-fold decrease in the number of CD8+ cytotoxic T-lymphocytes (T-cytotoxic cells; T_c_ cells) (p=0.0022, Mann-Whitney test, **Figure 1D**) in GK rats compared to control Wistar rats.

GK rats exhibit a ~2-fold increase in CD161a+ cells (p<0.0001, Mann-Whitney test, **Figure 1E**), a “target specific receptor”, which enables NK cells to activate and efficiently carry out their cytotoxic functions.

We also observed that despite the low percentage of T_c_ cells, majority of them were RORγT+ (p=0.0022, Mann-Whitney test, ~1.5-fold increase in GK rats, **Figure 1F**) and GATA3+ (p=0.039, Mann-Whitney test, ~1.5-fold increase in GK rats, **Figure 1G**) compared to Wistar rats. Furthermore, cell population frequencies of CD45RA+ RT1B+ peripheral B cells, Tregs (CD4+ CD25+ FoxP3+), Th1 cells (CD4+ Tbet+), Th2 cells (CD4+ GATA3+), Th17 cells (CD4+ RORγT+) and Tc1 cells (CD8+ Tbet+) were comparable between Wistar and GK rats (**Supplementary Figure 2**). The cell populations that were most highly increased in the GK rats were unidentified CD11b/c- CD45RA- cells (**Supplementary Figure 2**; p=0.0022; Mann-Whitney test). Thus, overall, GK rats show a very distinct immune profile that may be a direct consequence of the diabetic phenotype.

### Plasma Cytokine Profile of GK Rats Reflects the Low Percentage of the Respective Secretory Immune Cells

**Figure 2:**
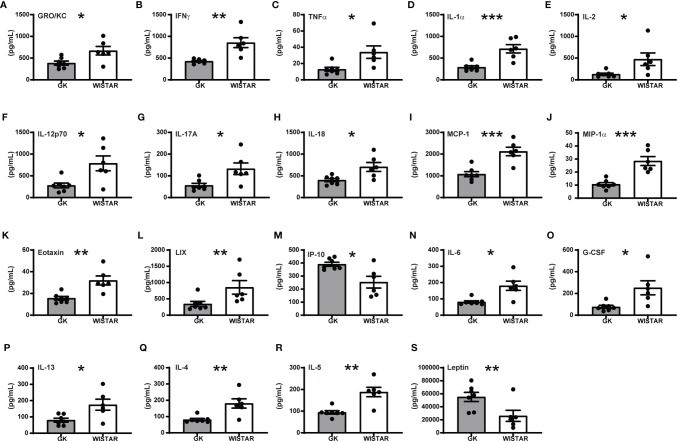
Overall cytokine profile shows limited peripheral inflammation in GK rats. All panels display the mean levels of plasma cytokines (mean +/- SEM) in pg/mL, that were significantly different (p<0.05) in the GK rats compared to the controls. Cytokines shown in panels **(A–M)** are proinflammatory, **(N)** is both pro- and anti-inflammatory, **(O–Q)** are anti-inflammatory while **(R, S)** show no inflammatory role. Grey bars: GK rats (n = 7); white bars: Wistar rats (n = 6). p < 0.001, p < 0.01 and p < 0.05 are reflected by ***, ** and * respectively. Student’s t-test was used except for panels E (IL-2), L (LIX), and R (IL5), which were analysed using a Mann-Whitney test.

Overall, our results revealed a strong impact of T2D on plasma cytokine levels. Surprisingly, most of the cytokines, regardless of its inflammatory role (pro- or anti- inflammatory) were significantly decreased in GK rats (**Figure 2**). Only 8 cytokines: EGF, Fractalkine IL-1β, IL-10, RANTES, VEGF, MIP-2 and GM-CSF levels were comparable in GK and Wistar rats (**Supplementary Figure 3**). Of the 16 pro-inflammatory cytokines investigated, levels of 13 were significantly lower (**Figures 2A–M**), in GK rats compared to Wistar rats (p-values between 0.0022 and 0.0115) as shown in **Figure 2**. The majority of these cytokines are secreted by myeloid-derived cells such as monocytes/macrophages including eotaxin, GRO/KC, IL-1α, IL-12(p70), LIX, MCP-1, TNF-α, and IL-18 (20–29). Furthermore, levels of cytokines secreted by lymphoid derived cells such as B-cells and T_c_ cells including IL-1β (30), MIP-1α (31) and IFN-γ (32), IL-2 (33) respectively were lower in GK rats. Similarly, levels of IL-17A was also found to be lower in the GKs. Unfortunately, this was not consistent with the increased number of circulating T_c_17 cells we observed. However, this can most probably be explained by reduced numbers of other primary sources of IL-17A such as T_h_17, NK cells, or natural killer T cells (34). Unlike other pro-inflammatory cytokines, IP-10 levels were significantly increased in GK rats (p=0.0103, **Figure 2M**).

IL-6 bridges anti- and pro-inflammatory actions and is primarily secreted by macrophages and monocytes. IL-6 levels were lower in GK rats **Figure 2N**; p<0.05). This was most likely due to the low percentage of macrophages/monocytes.

Three out of the four anti-inflammatory cytokines investigated (G-CSF, IL-13 and IL-4) were significantly decreased in GK rats (**Figures 2O–Q**, 0.0001<p<0.02). G-CSF is produced by monocytes and macrophages and their low levels in the GK plasma is consistent with our flow cytometry data (low percentage of CD11b/c+ cells). IL-13 is secreted by macrophages, B-cells and T_c_ cells, all of which show low frequency resulting in low secretion of these cytokines. IL-4 and IL-5 (**Figures 2Q, R**, Mann-Whitney test for IL-5) has been reported to be secreted by T_h_2 cells, basophils and eosinophils. Finally, leptin (**Figure 2S**) was the unique plasma marker that was significantly upregulated in GK rats compared to Wistar animals (p<0.001). Elevated levels of leptin could correlate with increased adipose depots in the GK rats, as we previously described (17).

**Figure 3:**
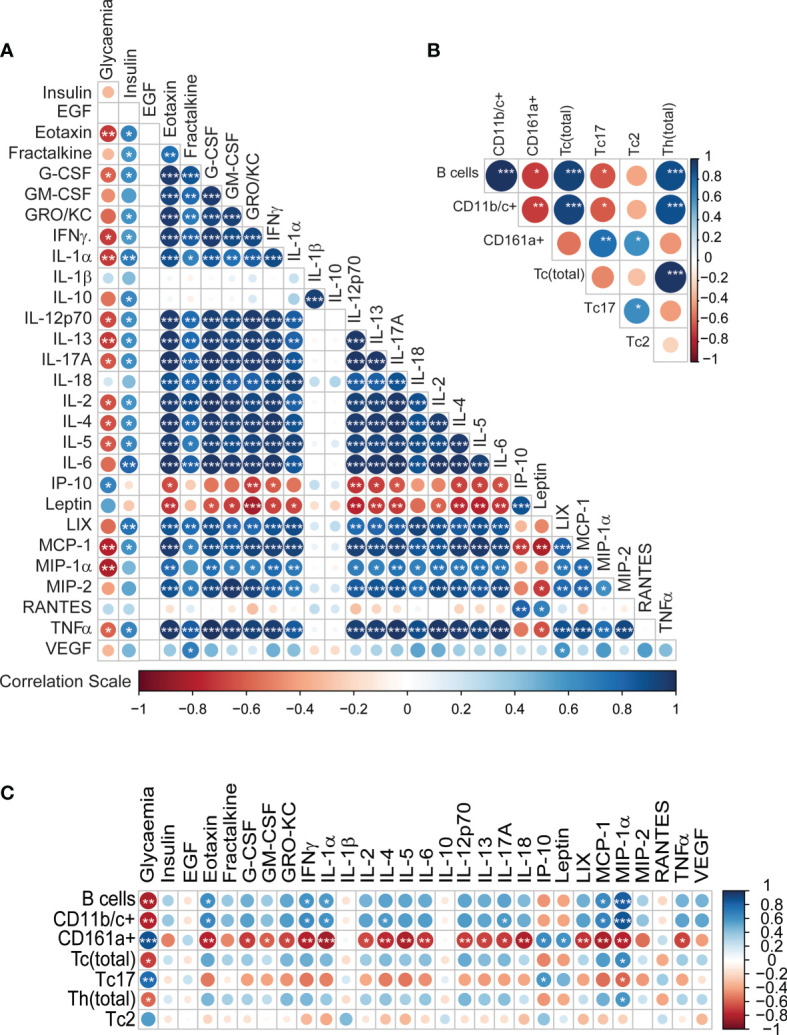
Integrative correlation analysis of the different immune parameters, **(A)** Cytokine-cytokine **(B)** Immune cell-immune cell **(C)** Cytokine-immune cells. The sizes of the circles indicate the strength of association (Pearson’s r^2^), while the colours show negative (red) or positive (blue) correlation. p < 0.001, p < 0.01 and p < 0.05 are reflected by ***, ** and * respectively.

Additionally, we also tested plasma glucose readings, which were ~2-fold (p<0.0001, Welch’s t-test) higher in GK rats compared to the controls (**Supplementary Figure 4**), while the insulin levels were comparable for the two groups. We then investigated correlations between the circulating cytokines and immune cells along with plasma readouts such as glycaemia and insulin levels. Bravais Pearson correlations revealed several significant associations between the metabolic markers and cytokine levels. Glycaemia was negatively correlated with the majority of cytokines levels (**Figure 3A**); whereas plasma insulin levels were positively correlated with cytokines levels (**Figure 3A**). Most of the cytokines were positively correlated each other; in contrast IP-10 and leptin were strongly negatively correlated with majority of the cytokines (**Figure 3A**). IL-1β levels were positively correlated with IL-10 levels but showed no association with any of the other cytokines (p<0.001). IP-10 correlations with cell numbers and cytokine levels are shown in **Supplementary Figures 5**, **6** respectively.

The correlation pattern for the immune cells was heterogeneous (**Figure 3B**). B-cells, CD11b/c+ cells, T_c_ cells and T_h_ cells, all positively correlated with one another (p<0.001). CD161a+ cells were positively correlated with T_c_17 and T_c_2 cells (0.01<p<0.05). CD161a+ cells and T_c_17 cells were negatively correlated with B-cells and CD11b/c+ cells (0.01<p<0.05). The associations between the immune cells and the cytokines also revealed some specific patterns (**Figure 3C**). Glycaemia showed a strong positive association with CD161a+ cells and T_c_17 cells; and negative correlation with B cells, CD11bc+ cells, cytotoxic T-cells and T_h_ cells. Interestingly, MIP-1α showed the exact opposite trend to glycaemia in terms of association. CD161a+ was negatively correlated with numerous cytokines. Thus, it is clear that the secretion pattern of most cytokines in our panel is dependent on the immune cell frequencies. Overall, the low levels of cytokines are consistent with the observed lower percentages of CD11b/c+ monocytes and macrophages, T_c_ cells and B-cells.

### GK Rats Show a Distinct Transcriptomic Profile in Key Diabetic Tissues, Which Also Affects Immune and Diabetic Biological Pathways

**Figure 4:**
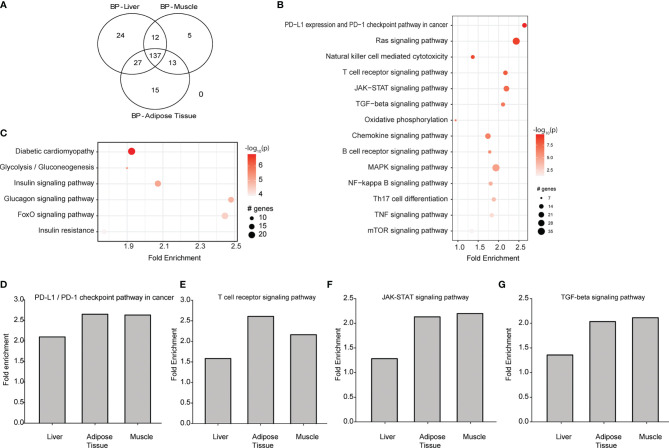
Transcriptomic re-analysis of 3 principal diabetic tissues (liver, muscle and adipose tissue) in Wistar rats compared to GK rats. **(A)** Venn diagram showing the number of common biological pathways. **(B)** Biological pathways that are regulated by downstream signalling from circulating cytokines analysed in the muscle. **(C)** Biological pathways associated with diabetes and its complications analysed in the muscle. For **(B, C)**, the intensity of the colour is proportional to the -log_10_ of P-value, sizes of the circles represent the number of genes involved, while placement of the circles on the x axis indicates the fold enrichment. **(D–G)** Are T-cell receptor signalling, Jak-STAT signalling, TGF-β signalling and PDL1 signalling respectively in liver, muscle and adipose tissue. Data from GSE13271 (16).

We re-analysed publicly available GK rat datasets and we observed 884, 665, and 1819 differentially expressed genes (DEGs; BH-adjusted p<0.05; log2 fold-change>2) in liver, muscle and adipose tissues respectively. Biological pathways were extracted from DEG profiles. Overall, we found 137 biological pathways common to all 3 tissues (**Figure 4A**). Of these, 14 were specific to downstream signalling from cytokines, and 6 were common diabetes-related pathways as shown in **Figures 4B, C** for muscles and **Supplementary Figure 7** for liver and adipose tissue. Cytokine dependent pathways such as T-cell receptor signalling, Jak-STAT signalling, TGF-β signalling, and PDL1 signalling (**Figures 4D–G**) were all significantly increased in Wistar compared to GK rats, consistent with the reduced circulating cytokine levels and numbers of cytokine secreting cells.

## Discussion

T2D has been commonly associated with low grade inflammation, but it is not clear whether this inflammatory profile is related to diabetes or to obesity. The aim of the present study was to examine peripheral inflammatory status in an animal model of T2D without obesity. Surprisingly, our results suggest that GK rats do not show a low-grade inflammatory profile as reported in obese models, but instead exhibit a marked decrease of the examined immune markers. Beta cell dysfunction in the GK rats is the primary defect that leads to overt hyperglycaemia and marked islet inflammation (13, 14, 35). Although, systemic inflammation is often associated with IR, it is not the sole contributor to the development of IR. Ycaza et al. reported that adipose insulin resistance did not correlate with markers of subcutaneous adipose inflammation (36). GK rats develop hepatic insulin resistance at a young age (37), while whole-body insulin resistance (which is not associated with liver, adipose or muscle inflammation) appears later in life (14). Pitasi et al. recently showed increased levels of GSK3B protein in the GK pancreas (14), which has been reported to be elevated in insulin target tissues in humans with T2D, and (38) is correlated with decreased insulin sensitivity (39). Thus, we postulate that a possible over expression of GSK3 in insulin target tissues in the GK rat, could participate to the development of insulin resistance in this model.

Here, we provide a detailed description of the GK rat immune system, which shows limited peripheral inflammation, in addition to a lower percentage of phagocytes (macrophages, monocytes, dendritic cells, granulocytes and B-cells). This partially confirms impaired phagocytosis and antigen presentation as previously described in GK rats (5, 10, 11), however Zhai et al. reported a higher percentage of activated B cells in obese diabetics, which does not fit our observation (40), suggesting a major role of obesity in the inflammatory profile. The observed low percentage of T_h_ cells in the GK rats may also have an effect on the activation status of B- and cytotoxic T-cells, and may further explain their lower numbers in GK rats. Diabetes has also been known to disrupt monocyte recruitment to sites of injury, thereby impairing phagocytosis and impeding the switch from pro- to an anti-inflammatory state (41). Interestingly, wound healing in diabetics is also known to be hindered as diabetes prompts neutrophils to cause tissue damage by NETosis (42), which further contributes to the inflammation causing a vicious pathological cycle (43).

We have seen a decrease in CD8+ T_c_ cells in our GK rats, which are normally responsible for eliminating viral-infected host cells and thus is also in line with the predisposition of T2D patients towards severe infections (44). This agrees with the reported decrease in naive T_c_ cells, and accumulation of EMRA immunosenescent cells in man (45).

A higher CD4/CD8 ratio conventionally corresponds to a healthier immune system and estimates the likelihood of development of infections. In rats, this ratio is strain specific (46). GK rats show a significantly higher CD4/CD8 ratio (**Supplementary Figure 8**), which can be attributed to the low number of T_c_ cells and may not necessarily represent a healthier immune system compared to Wistar rats. Interestingly, a recent study also reported a higher CD4/CD8 ratio after a glucose bolus in both diabetics and non-diabetics (47), indicating a lymphocyte redistribution, which may be the case for our GK rats as well. We also examined T_h_1/T_h_2, T_c_1/T_c_2 and T_h_17/Treg ratio, which have been reported/reviewed to be impaired in T2D with comorbid obesity (48), inflammatory diseases such as Behcet’s disease (49) and metabolic disorders (50) respectively. We did not see significant differences for any of these ratios in our GK rats (p>0.05, Mann-Whiney test, data not shown).

We interpret the overexpression of CD161a in GK rats as most likely to be an already activated defence system as a compensatory mechanism for their compromised immune system to combat an invading or existing pathogen/infection. It is logical to conclude that this is achieved by the activation of monocytes and NK cells to target infected cells and by secretion of cytokines to further activate these cells or this can be indicative of healthy cell/tissue destruction. Altered immune functionality in both human B- and NK-cells has been reported to be mediated by global hypermethylation in obese (BMI>30) T2D patients (51). The data on human NK cells is somewhat unclear. Meta-analysis of 13 NK cell studies identified 3 with decreased levels/activity, 2 reported elevated levels and 8 of them reported no differences between circulating NK cells in T2D patients and healthy controls (52).

RORγT+CD8+ lymphocytes are a somewhat underexplored population, but they are very pro-inflammatory (53). GK rats show pancreatic islet inflammation (35) and in our data, peripheral inflammation appears to be limited to the observed increase in T_c_17 cell population. T_c_17 cells are produced under inflammatory conditions in a manner similar to their CD4+ counterparts, T_h_17 cells (54), skewing the immune system towards a pro-inflammatory phenotype, although, as outlined above IL-17 levels are lower in GK rats. Interestingly, CD8+T_c_17 cells have been reported to show a strong repression of *SOCS3* expression (55), a pivotal gene regulating insulin sensitivity (56) in addition to driving inflammation [as reviewed (57)]. T_c_17 cells have been shown to degrade pancreatic beta islet cells and induce hyperglycaemia in mice (57). Thus, it is quite likely that this cell population contributes to the vicious diabetic and (peripheral) inflammatory aetiopathology of the GK rats.

Similarly to the T_c_17 cells, T_c_2 cells have not been highly investigated in the light of diabetes but have been reported to be more diabetogenic compared to naive T_c_ cells (58). However, in T2D with comorbid tuberculosis, there appear to be a clear role for both T_c_17 and T_c_2 cells (59). Although, there is a preponderance of literature on immune changes in complicated diabetes and unfortunately, the immune status in uncomplicated human T2D is still ambiguous.

Circulating baseline cytokine levels have been studied in T2D for many decades (60). In this study, we present the first account of multiplexing a panel of 27 cytokines simultaneously from the same samples to get a clearer, detailed overall picture of the GK immune system (**Figure 2**). The reduced levels of the cytokines in GK rats is most likely due to low number of circulating CD11b/c+ cells, principally monocytes/macrophages in addition to low percentages of CD45RA+ (B-cells) and CD8+ (T_c_ cells) cell populations. Interestingly, despite an overall decrease of both anti- and pro-inflammatory cytokines plasma levels, our results reveal a significant increase of interferon gamma-induced protein 10 (IP-10), a pro-inflammatory chemokine which plays an important role in the aetiology of inflammatory diseases. Previous studies suggest that IP-10 is associated with metabolic disorders. Accordingly, IP-10 levels were increased in the early stage of type 1 diabetes (61) and were also predictive of insulin resistance and diabetes in patients suffering from nonalcoholic fatty liver disease (62). Previous studies suggest a positive association between leptin levels and IP-10 in type 2 diabetic patients (63), which also fits our GK rat data (**Supplementary Figure 5V**).

We also saw an increase in leptin levels in our GK rats. However, this does not necessarily indicate an increased secretion of proinflammatory cytokines from GK adipocytes due to tissue specific gene expression. Previously, we have shown that the expression of cytokines such as IL-6, IL-1β and TNF-α were increased in the GK islets, but not in the adipose tissues (14). Thus, while we observe elevated levels of adipokines associated with the increased adiposity in GK rats, we do not see an increased pattern secreted proinflammatory cytokines.

Thus, it is clear from our study that the GK cytokine profile alone can be quite misleading, unless amalgamated with cognate immune date. However, there is no clear pro-inflammatory cytokine profile as previously thought. Our data suggests that the inflammatory milieu in GK rats is most likely due to T_c_17 cells and activated monocytes, NK cells as represented by elevated CD161a+ cells. In the human context, T2D patients have been shown to have higher IL-17 levels compared to healthy controls, which contribute to the pro-inflammatory phenotype commonly observed in T2D (64, 65), thereby justifying our hypothesis.

This disproof of the proinflammatory profile in GK rats led us to investigate the gene expression pattern in liver, muscle and adipose tissues: all key tissues that not only play important roles in glucose homeostasis affecting glycaemic status but are also exposed to the circulating immune cells and cytokines. Therefore, we re-analysed previously published GK rat micro-array expression data for these three key diabetes related tissues. The extracted biological pathways from the gene expression profiles confirmed a downregulated pattern of most involved genes in the GK rats. The most prominent gene pathway was diabetic cardiomyopathy, fitting previous reports of ventricular hypertrophy, impaired diastolic function, and cardiomyopathy in GK rats (66, 67) that may be mediated by CRP (68). This peripheral gene expression profile contrasts sharply with the inflammatory profile of the GK rat pancreatic islets, where similar to diabetic human islets (69), we consistently show increased pro-inflammatory cytokine levels, associated with increased pro-inflammatory immune cell numbers around and within the pancreatic islets (13, 14, 35). This profound islet inflammation is a contributor to the impairment of beta cell growth and function, which constitutes the main characteristics of T2D in this model. GK rats serve as an excellent model to study the development of T2D, and the transition from prediabetic to a fully diabetic phenotype may yield insight into the development of T2D.

In conclusion, our study reports 3 lines of evidence proving that, outside the pancreatic islets, GK rats present a limited pro-inflammatory profile unlike previously thought. Additionally, non-obese GK rats exhibit a marked immune dysfunction as indicated by the overall blunted levels of cytokines and changes in the distribution of immune cells. Thus, it is also clear that there is a knowledge gap between uncomplicated human non-obese T2D, and the immune system that needs to be addressed in detail and that GK rats can serve as a good model for this.

## Data Availability Statement

The raw data supporting the conclusions of this article are available from the corresponding author upon reasonable request.

## Ethics Statement

All experimental procedures were carried out in accordance with the European Union guidelines for the use of animals for experimental purposes (Council Directive 2010/63/EU) and with the French ones (Directive 87/148, Ministère de l’Agriculture et de la Pêche – Apafis #16924).

## Author Contributions

Conceptualization, SS, MD, and JT. Data collection, MH, CP, SS, DB, JM, and MD. Data analysis, SS, CH, MH, CP, MD, JT. Writing and editing, all authors. All authors contributed to the article and approved the submitted version.

## Funding

This work was funded by French National Research Agency (ANR-17-CE37-0020, Maternal diabetes and neuropsychiatric vulnerability in offspring: role of DNA methylation -MADAM) and by Fonds National de Recherche Luxembourg (INTER/ANR/16/11568350 ‘MADAM’) and (Age Acceleration through the life course (C19/SC/13650569 ‘ALAC’). This work was supported by the University of Bordeaux and by the AlimH department of the Institut National de la Recherche Agronomique (INRAE).

## Conflict of Interest

The authors declare that the research was conducted in the absence of any commercial or financial relationships that could be construed as a potential conflict of interest.

## Publisher’s Note

All claims expressed in this article are solely those of the authors and do not necessarily represent those of their affiliated organizations, or those of the publisher, the editors and the reviewers. Any product that may be evaluated in this article, or claim that may be made by its manufacturer, is not guaranteed or endorsed by the publisher.
