# Supplementary material for: A Holistic View of the Goto-Kakizaki Rat Immune System: Decreased Circulating Immune Markers in Non- Obese Type 2 Diabetes
Source: Front Immunol. 2022 May 23;13:896179. doi: 10.3389/fimmu.2022.896179 (PMC9168276; doi:10.3389/fimmu.2022.896179)

A holistic view of the Goto-Kakizaki rat immune system: type-2 diabetes and the pro-inflammatory milieu in the absence of obesity

Snehaa V. Seal[1,2], Mathilde Henry[3], Clementine Pajot[3], Cyrielle Holuka[1,2], Danielle Bailbé[4], Jamileh Movassat[4], Muriel Darnaudéry[3#], Jonathan D. Turner[1*#]

Supplementary Figure 1: Flow cytometry gating strategy used to investigate the frequencies of the immune cell populations. The data are expressed as percentage of frequency of parent population. The pink and the purple boxes show the frequencies of the T-helper and T-cytotoxic cell subsets respectively.

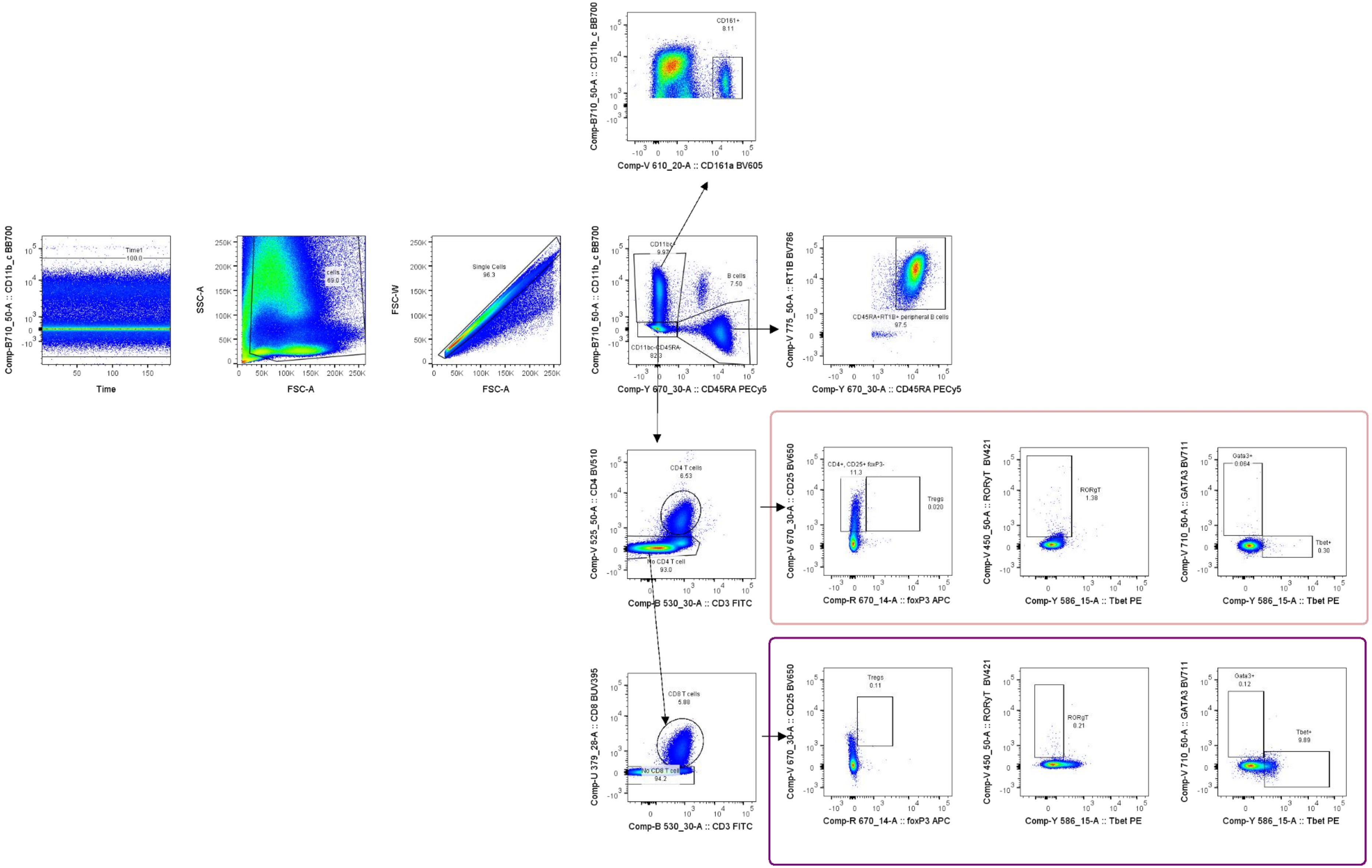

Supplementary Figure 2: Immune cell populations that were not significantly different between the GK and Wistar rats (from the same panel as Figure 1), displayed as means along with their SEM in frequency of parent (%). The colours of the bars represent the different strains (GK rats = grey, Wistar rats = white). Mann-Whitney test was used for all panels.

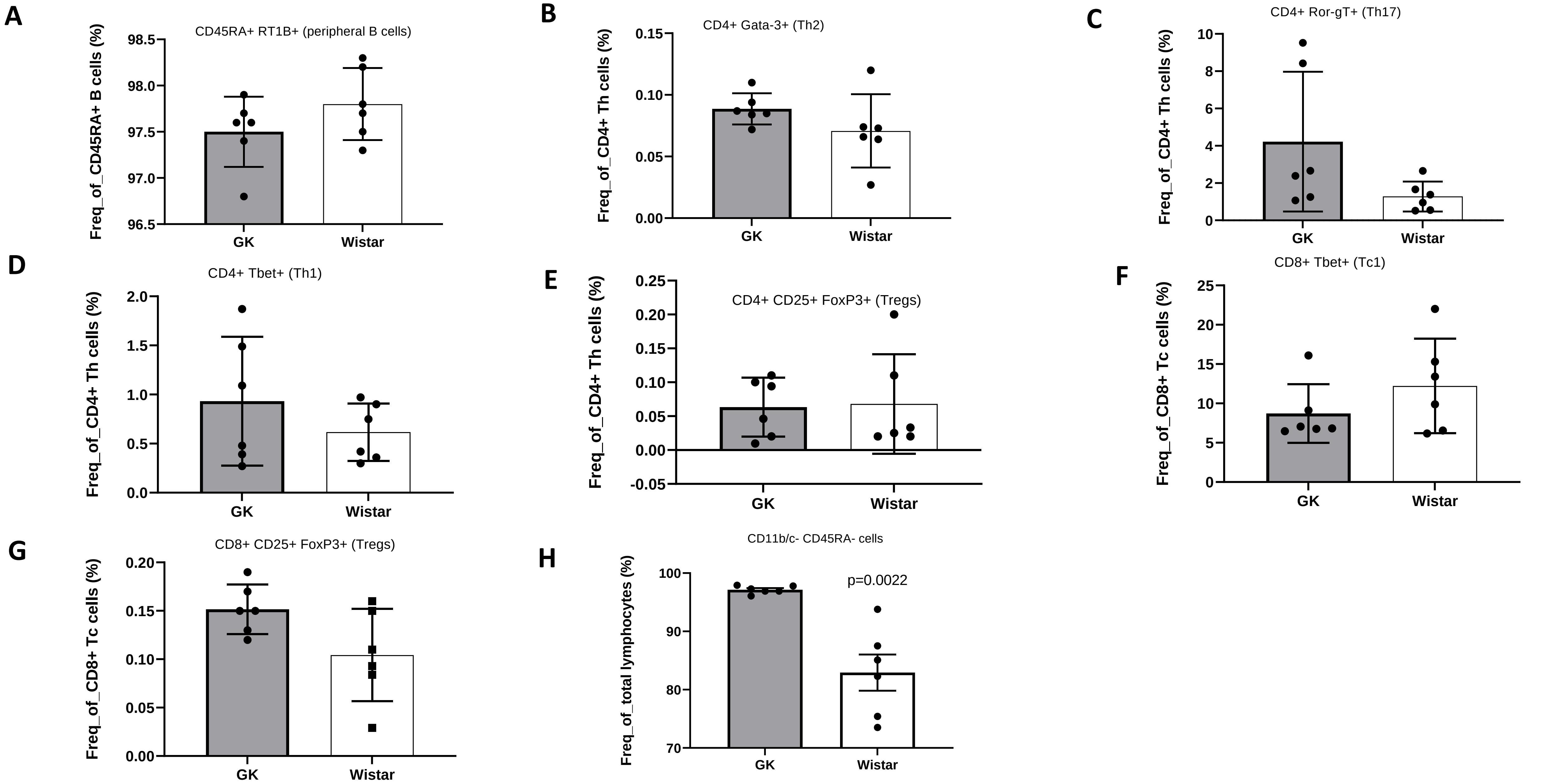

Supplementary Figure 3: Cytokines that were not significantly different between the GK and Wistar rats (from the same panel as Figure 2), displayed as means along with their SEM in pg/mL. Cytokines shown in Figures A-C are proinflammatory, D) is both pro and anti-inflammatory, E) is anti-inflammatiory and F-H show no inflammatory role. The colours of the bars represent the different strains (GK rats = grey, Wistar rats = white). Student’s t-test was used except for panels H (EGF) and A (MIP-2).

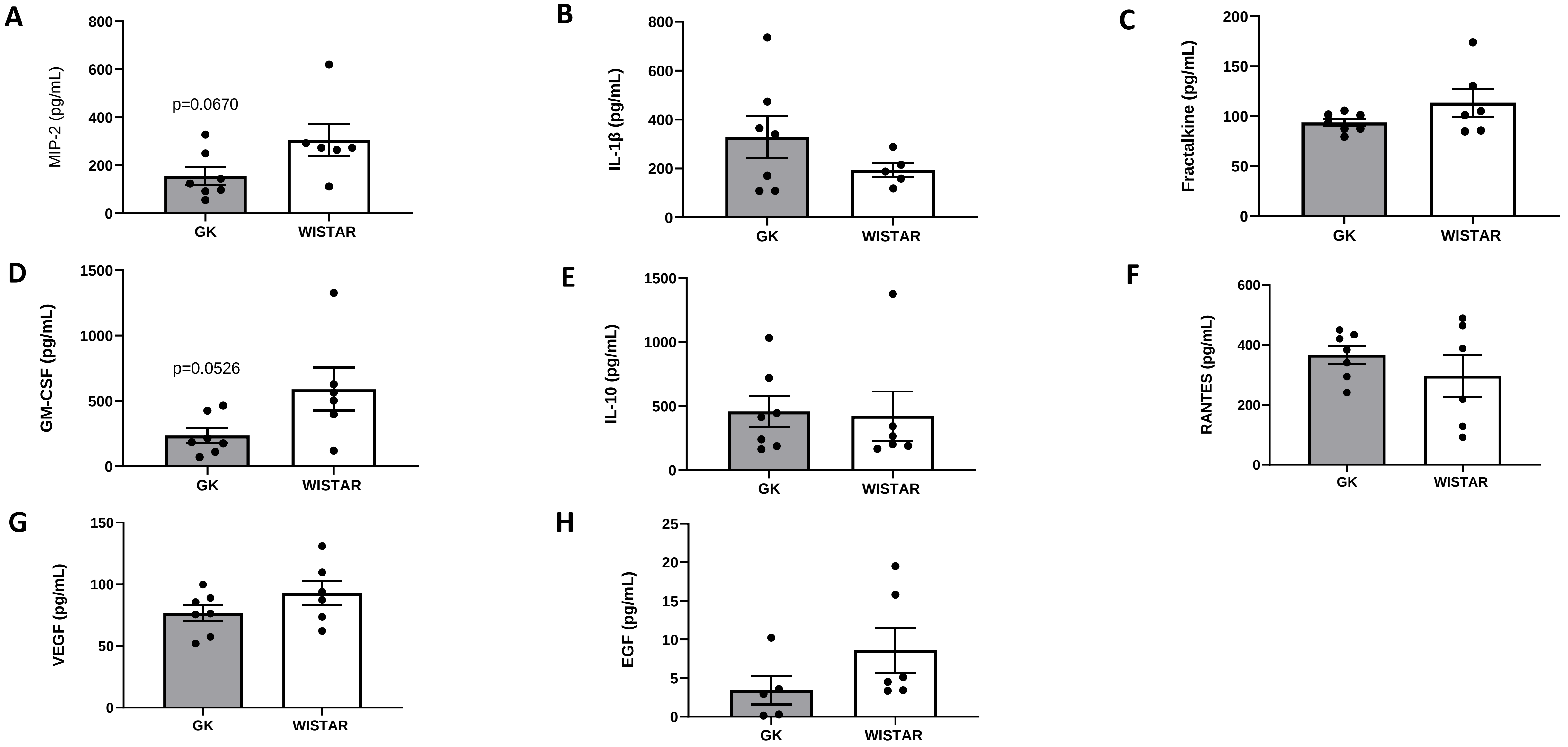

Supplementary Figure 4: Plasma readouts for GK rats. Panels A) and B) show glucose and insulin levels in GK rats compared to control Wistar rats respectively. Data are mean+/- SEM and the colours of the bars represent the different strains (GK rats = grey, Wistar rats = white).

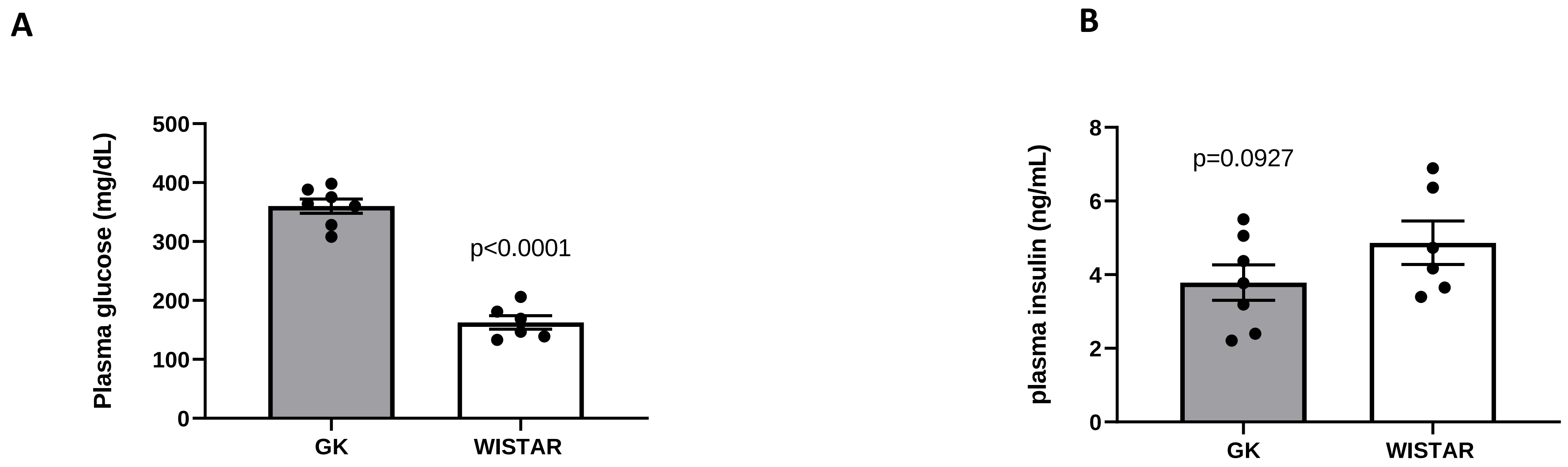

Supplementary Figure 5: Correlation scatter plots (A-N) show no correlation between levels of IP-10 (pg/mL) and immune cells (frequency of parent population) between GK and Wistar rats. GK rats = black dots, Wistar rats = empty dots.

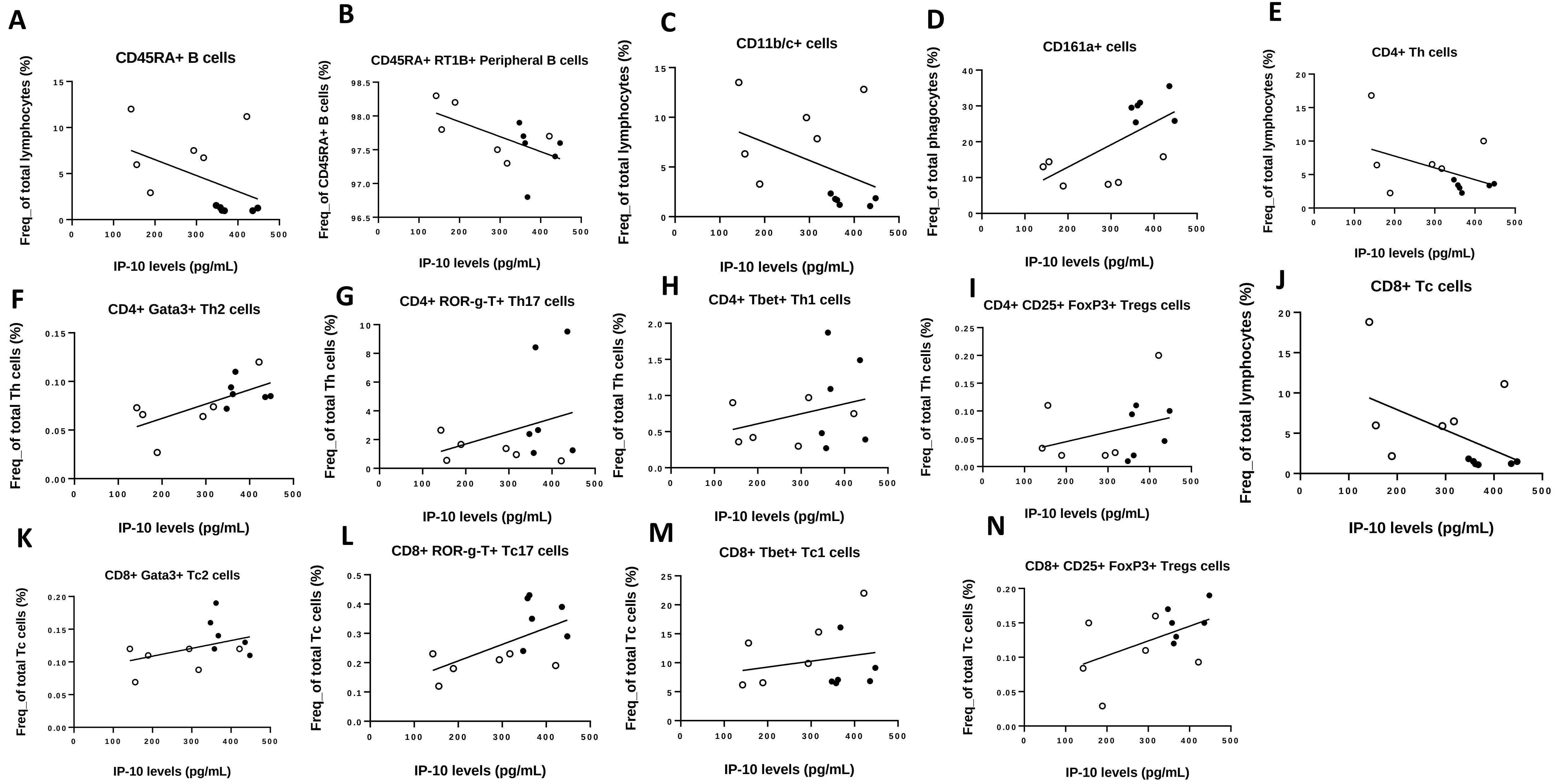

Supplementary Figure 6: Correlation scatter plots (A-N) show no correlation between levels of IP-10 (pg/mL) and other cytokines (pg/mL) between GK and Wistar rats. GK rats = black dots, Wistar rats = empty dots. Cytokines shown in Figures A-O are proinflammatory, P-Q) are both pro and anti-inflammatory, R-U) are anti-inflammatiory and V-z show no inflammatory role.

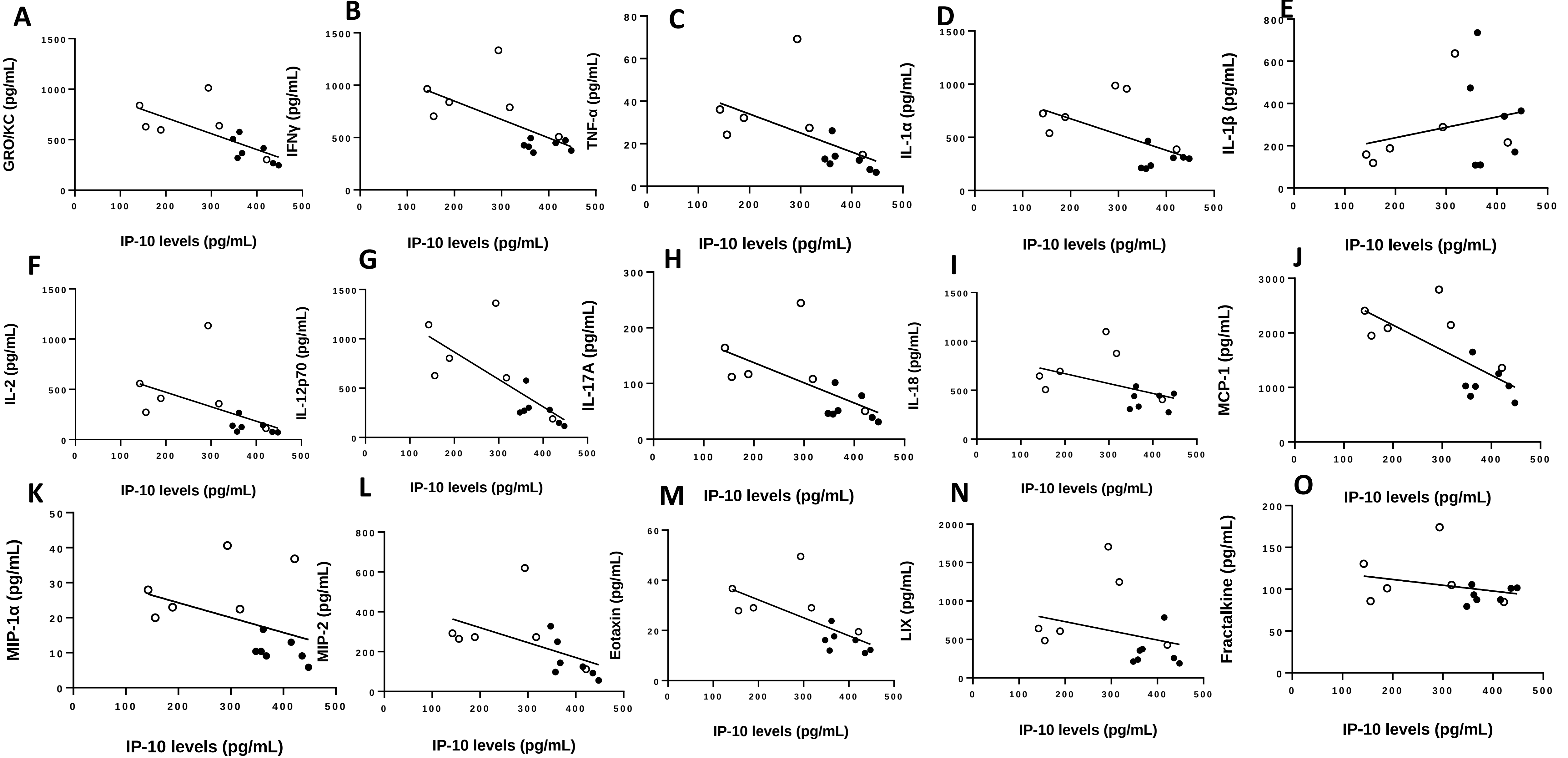

Supplementary Figure 6 continued: Correlation scatter plots (A-N) show no correlation between levels of IP-10 (pg/mL) and other cytokines (pg/mL) between GK and Wistar rats. GK rats = black dots, Wistar rats = empty dots. Cytokines shown in Figures A-O are proinflammatory, P-Q) are both pro and anti-inflammatory, R-U) are anti-inflammatiory and V-z show no inflammatory role.

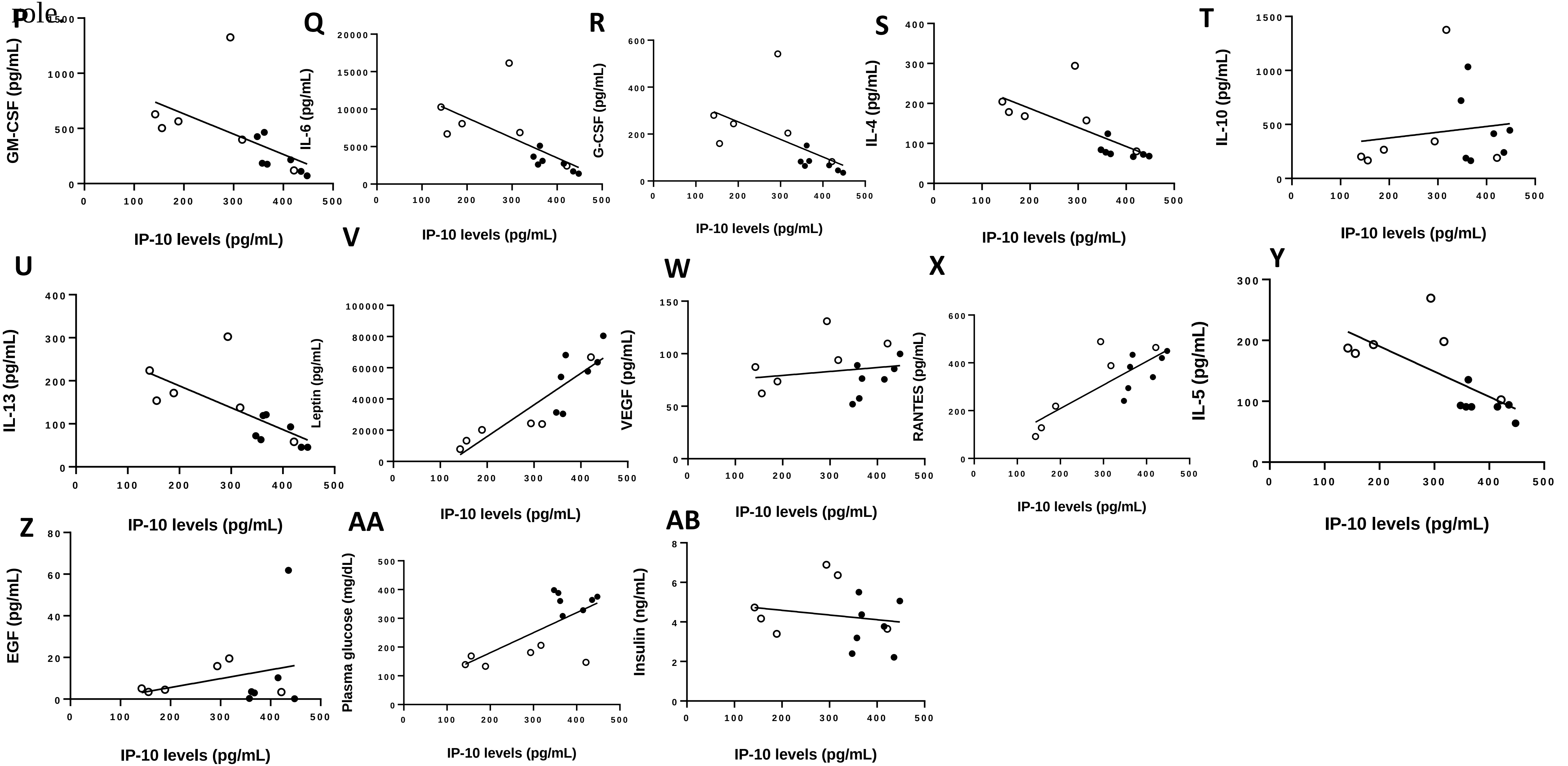

Supplementary Figure 7: Transcriptomic analysis in Wistar rats compared to GK rats. Panels A) and B) show pathways regulated by downstream signalling from circulating cytokines analysed in the liver and adipose tissue respectively.

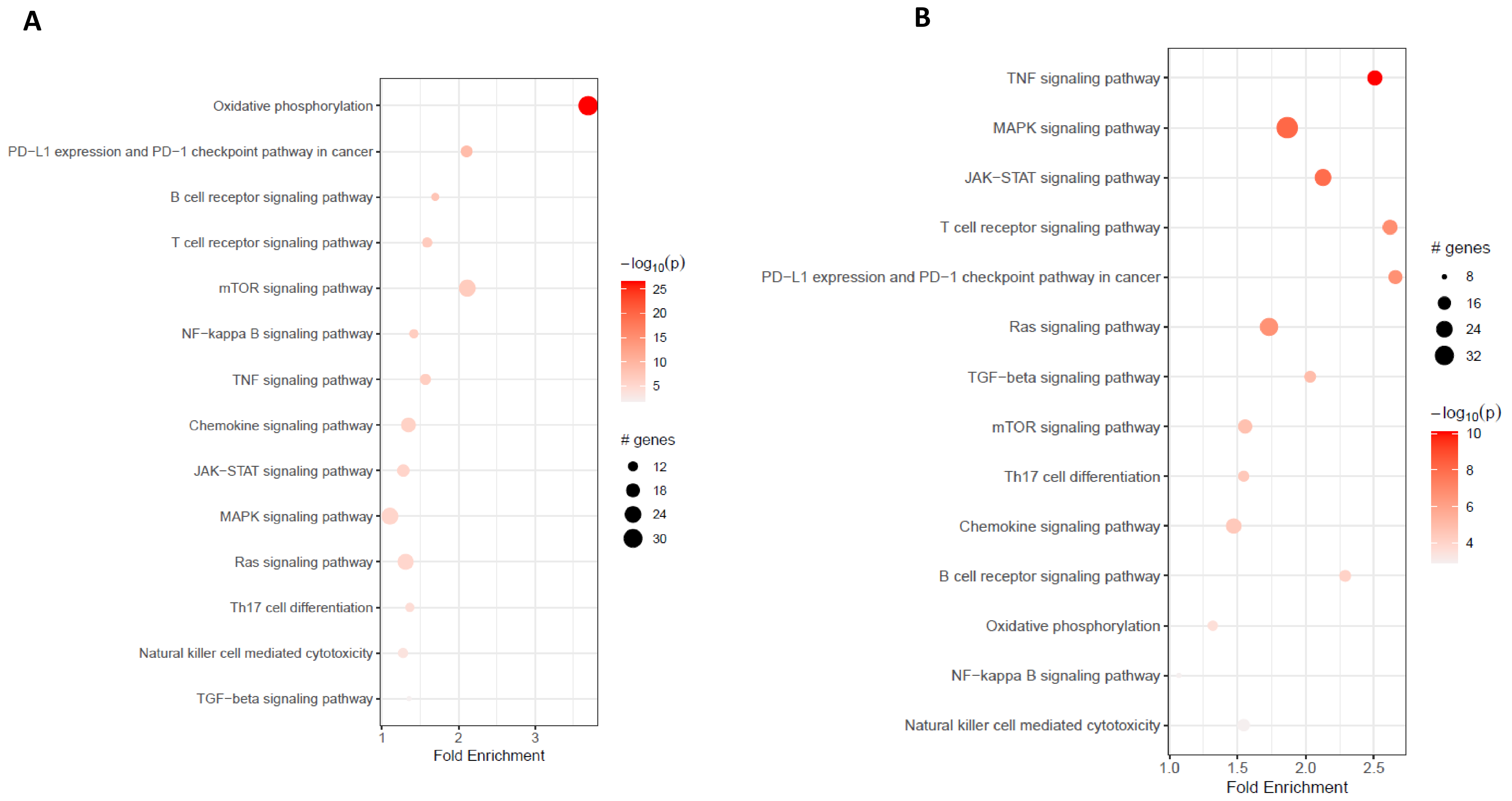

Supplementary Figure 7 Continued: Transcriptomic analysis in Wistar rats compared to GK rats. Panels C) and D) show pathways associated with diabetes and its complications analysed in the liver and adipose tissue respectively. Data from GSE13271.

C

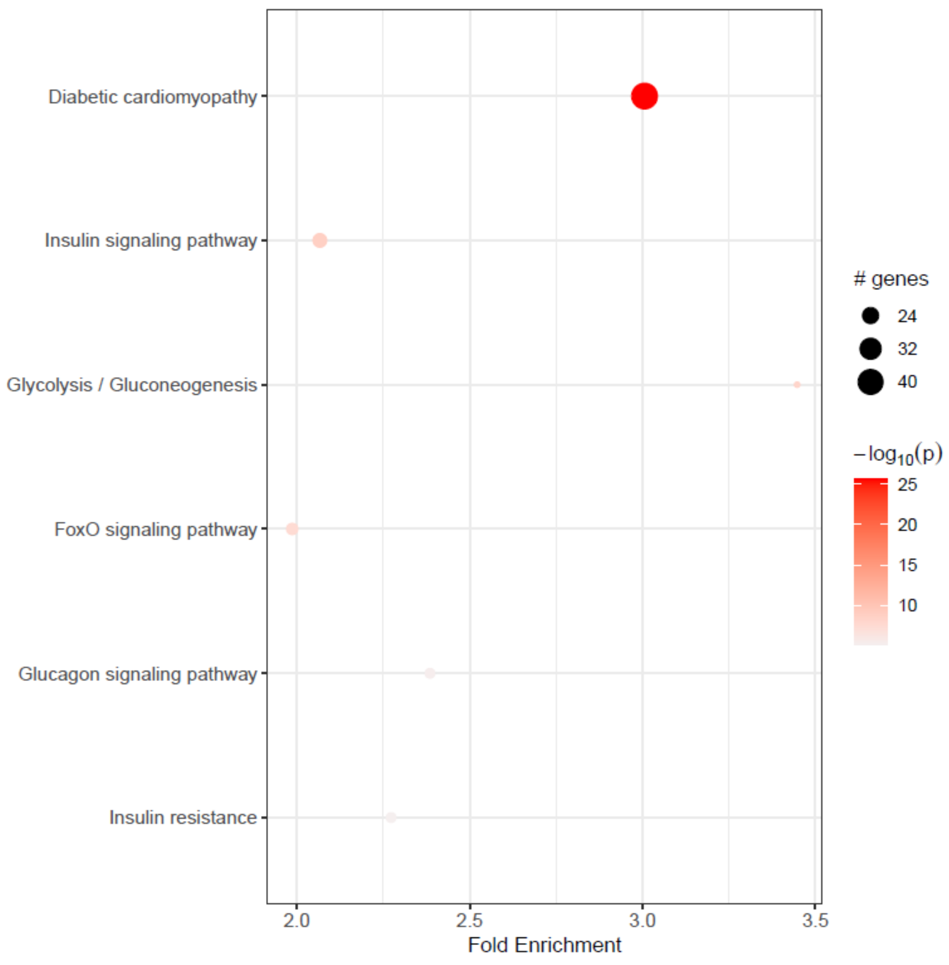

D

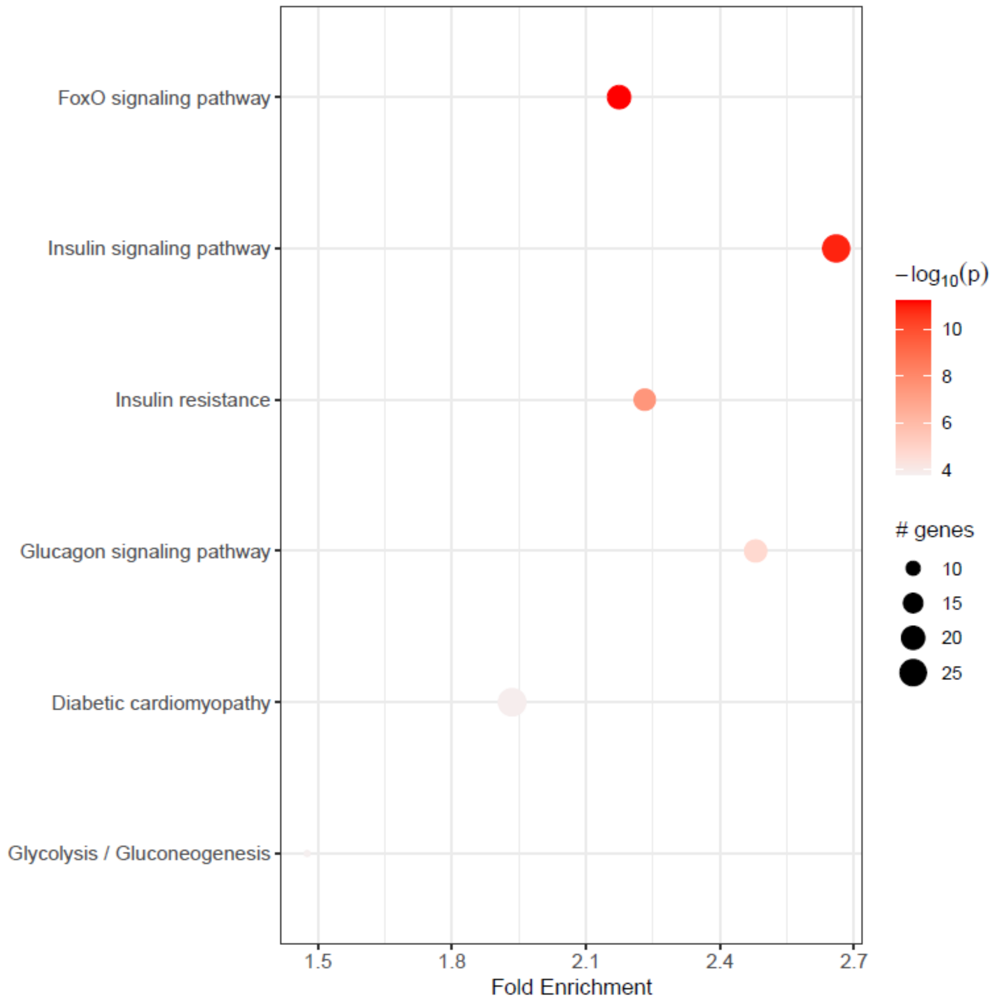

Supplementary Figure 8: Ratio of CD4/CD8 cells shown as means along with the SEM. The colours of the bars represent the different strains (GK rats = grey, Wistar rats = white).

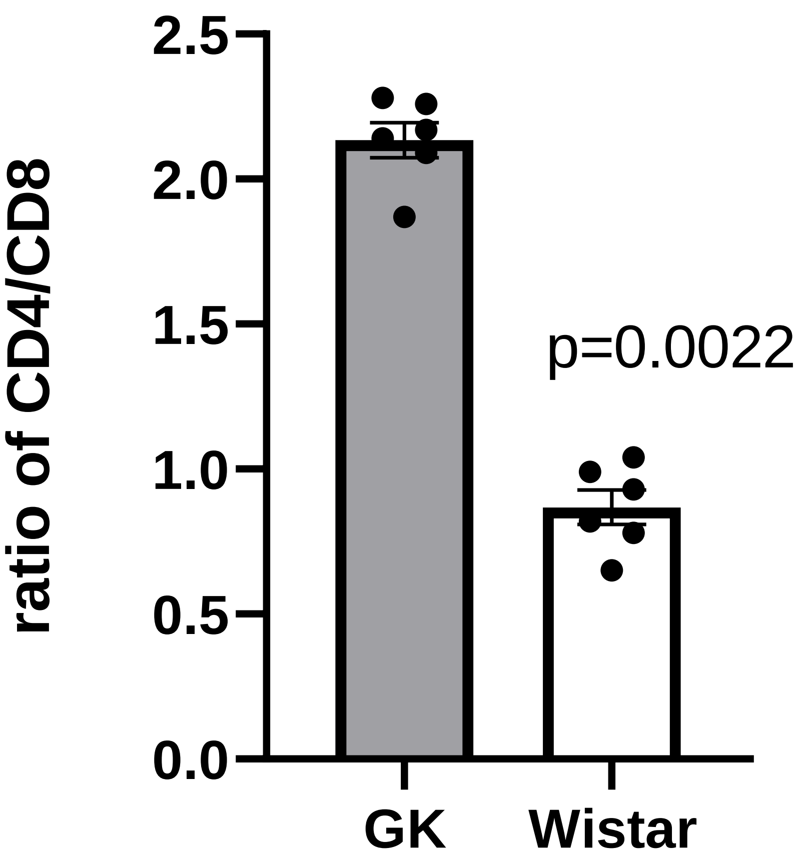

Supplement: Supplementary file 1 [file DataSheet_1.pdf]
